# Association of perceived neighborhood air quality problems with attempt to quit cigarette smoking: a cross-sectional study in Texas

**DOI:** 10.3389/fpubh.2024.1392065

**Published:** 2024-07-26

**Authors:** Monalisa Chandra, Joel Fokom Domgue, Robert Yu, Sanjay Shete

**Affiliations:** ^1^Department of Epidemiology, The University of Texas MD Anderson Cancer Center, Houston, TX, United States; ^2^Division of Cancer Prevention and Population Sciences, The University of Texas MD Anderson Cancer Center, Houston, TX, United States; ^3^Department of Biostatistics, The University of Texas MD Anderson Cancer Center, Houston, TX, United States

**Keywords:** cigarette smoking, attempt to quit smoking, air quality, neighborhood, Texas

## Abstract

**Background:**

Cigarette smoking is the major preventable cause of premature deaths in the United States. Attempting to quit smoking is an important step toward smoking cessation. Although it has been studied extensively, limited information on the association between attempts to quit smoking and neighborhood air quality problems is available. Therefore, we examined the association between attempts to quit smoking in the past year and perceived neighborhood air quality problems among adult Texans who smoke.

**Methods:**

In 2018, a cross-sectional multistage area probability design-based survey was administered to collect sociodemographic, behavioral, and health-related information from a representative sample of 2050 Texas residents. The current study included 486 adult respondents who reported smoking within the past 12 months. The association between attempts to quit smoking and perceived neighborhood air quality (measured by self-reported problems with neighborhood air quality) was examined using a population-weighted multivariable logistic regression analysis.

**Results:**

Overall, 60.7% of the 486 respondents attempted to quit cigarette smoking. The prevalence of attempting to quit was 74.6% for those reporting perceived neighborhood air quality problems. In the multivariable analysis, a higher likelihood of attempting to quit smoking was found among individuals with perceived neighborhood air quality problems (AOR: 1.906 [1.104–3.289]) and those who were married or living as married (AOR: 1.876 [1.161–3.033]). The likelihood of attempts to quit smoking was lower among males (AOR: 0.629 [0.397–0.995]) and decreased with age (AOR: 0.968 [0.951–0.984]).

**Discussion:**

The perceived neighborhood air quality problems were found to independently predict attempts to quit cigarette smoking in Texas. To encourage quitting smoking among individuals living in neighborhoods with poor air quality, such neighborhoods should receive tailored and evidence-based interventions to improve community education, social support, and healthcare professionals’ assistance to quit smoking.

## Background

Cigarette smoking is the leading cause of preventable diseases in the United States (1). In Texas, despite the decline in adult cigarette smoking over recent years (19.2% in 2014 to 13.1% in 2021) (2), its prevalence was higher than the national average (11.5% in 2021) (1). Moreover, 28,000 adults die each year in Texas due to smoking-related diseases (3). Quitting smoking reduces adverse outcomes such as cancer, cardiovascular diseases, and chronic obstructive pulmonary diseases (4). An attempt to quit smoking is an important step toward smoking cessation. However, 53.9% of adults attempted to quit smoking, and an even smaller proportion (8.9%) were successful at quitting in the United States in 2020 (5).

Based on the transtheoretical model, a health behavior change happens through multiple stages: pre-contemplation, contemplation, preparation, action, maintenance, and termination (6). A measurable behavioral change in the smoking cessation process is first observed at the *action* stage when individuals attempt to quit smoking (6). Thus, this stage is crucial in the process of embarking individuals on the path to successful smoking cessation. Attempts to quit smoking are primarily found to be associated with individual-level factors such as age, race (7), socioeconomic status (8, 9), healthcare professional’s advice to quit smoking (10), access to cognitive and pharmacological interventions (4), social support (11, 12), knowledge (13), self-efficacy, motivation, intent to quit, and depression (14, 15). The role of neighborhood characteristics on quit attempts and smoking cessation has gained more attention since the mid-2000s, with the rising relevance of contextual factors on individual well-being. Studies have found that adverse neighborhood characteristics were associated with increased stress, negative affect, disrupted social ties (16), and weakened sense of agency (17), which have implications for individual health behavior.

Neighborhood studies have shown that area-level socioeconomic attributes and household structure were associated with quitting smoking (18). Perceived neighborhood deprivation (smell/stench, noise, and criminal activities in the neighborhood), perceived physical conditions (neighborhood cleanliness, noise, traffic, and house conditions), and social disorder (safety, trust, and protection) were associated with a decreased likelihood of cigarette smoking abstinence (18–20). However, these reports did not study the association between quit attempts and neighborhood air quality. High exposure to poor air quality is responsible for multiple comorbidities such as chronic obstructive pulmonary disorder (COPD), cardiovascular disorder (CVD), asthma, cancer, and poor birth outcomes (21–23). Studies have also found that people with existing comorbidities, such as COPD and diabetes, are at heightened risk of worsening health conditions when exposed to particulate matter in the air (24, 25). While smoking, in general, is detrimental to individuals with comorbidities (26–28), it was found that smoking modifies the relationship between lung function and the level of NO_2_ and PM_2.5_ (air particulate matter) exposure in the ambient air with a stronger adverse association for current smokers (29). Therefore, a current smoker living in a neighborhood with poor air quality and struggling with reduced lung capacity will have greater motivation and perceived control in attempting to quit smoking than cleaning the ambient air.

However, to the best of our knowledge, no information is available on how an attempt to quit smoking is affected by the poor air quality in the neighborhood. In this study, we examined the role of perceived neighborhood air quality on the prevalence of attempts to quit smoking. We hypothesized that individuals with perceived neighborhood air quality problems would be more likely to attempt to quit smoking than those with no perceived neighborhood air quality problems.

## Materials and methods

### Participants and procedures

Our study was based on data from a cross-sectional multistage area probability design-based survey administered through Qualtrics restricted to opt-in panelists living in Texas to collect sociodemographic, behavioral, and health information. The survey was conducted between 5th February 2018 and 5th March 2018, and it was administered in English and Spanish (with translation performed by Master World Services Inc., Houston, TX, United States) via Qualtrics. A target was set for strata by sex (50% each for male and female), annual household income (48%, < $50,000; 30%, $50,000–$99,999; and 22%, ≥ $100,000), ethnicity/race [34% Hispanic/Latinos, 36% Non-Hispanic Whites (NHW), 25% Non-Hispanic Blacks (NHB), and 5% Asian/other], and locality (60% urban and 40% rural). Oversampling of certain strata was undertaken to increase estimation accuracy in those subgroups. Participants affirming Mexican, Hispanic, or Latino ethnicity were categorized as Hispanics regardless of race. Those selecting White as the sole race and non-Hispanic ethnicity were assigned to the NHW category. Those checking Black/African Americans alone or with other races and checked non-Hispanic ethnicity were categorized into the NHB category. Rural–urban residence was defined by matching ZIP codes to county designations. The participants received compensation of up to $10. After a pilot launch of the first 50 participants, the survey was implemented with a speed checker, with a final sample of 2050 complete responses (30, 31).

The current study was based on the data from adult Texans who reported having smoked in the past year (*N* = 486). The study population was screened for eligibility based on the question, “Do you now smoke cigarettes?” The response options were “Everyday,” “Somedays,” and “Not at all.” Those who responded “Everyday” and “Somedays” were included in the study population. Those who responded, “not at all” were asked another question, “About how long has it been since you completely quit smoking cigarettes?” The response was expected to be either in the number of “days,” “months,” or “years” of quitting. Those responding >365 days, >12 months, or > 1 year were excluded from the study. Our study followed Strengthening the Reporting of Observational studies in Epidemiology (STROBE) guidelines. Informed consent was obtained from participants, and the study was approved by the University of Texas MD Anderson Cancer Center’s Institutional Review Board (PA16–0724).

### Outcome measures

The outcome variable (attempt to quit cigarette smoking) was measured using the question, “At any time in the past year, have you stopped smoking for 1 day or longer because you were trying to quit?” The response to this question was recorded as “yes” or “no.” Those who responded “yes” were categorized as “attempted to quit.”

### Primary predictor

Perceived neighborhood air quality problem was the primary predictor of the study. The variable was measured using the question, “Please tell me whether or not each of the following is a problem in your community or neighborhood,” and the problems measuring air pollution in the neighborhood included in the survey were (1) “Fumes, smells, and smoke from traffic”; (2) “Fumes, smells, and smoke from industry.” Possible response options for each question were “yes” and “no.” The variable “perceived neighborhood air quality problem” was coded “yes” when the response to either question (1) or (2) was yes, and it was coded “no” when the response to both questions was “no.”

### Covariates

The analysis was adjusted for other factors, including healthcare professionals’ advice to quit smoking, as well as sociodemographic factors such as age, sex, race/ethnicity, marital status, household income, and occupation.

Healthcare professionals’ advice to quit smoking was measured using the question, “In the past 12 MONTHS, has a medical doctor, dentist, or other health professional ADVISED you to quit smoking, or to quit using other kinds of tobacco?” The possible responses were “yes” or “no.”

Other covariates included in the analysis were demographic and socioeconomic factors. Age was included as a continuous variable, and sex (Male/Female), race/ethnicity [Non-Hispanic Whites (NHW)/Non-Hispanic Black (NHB)/Hispanic/others], marital status (Single, Widowed, Divorced, Separated/Living as married, married), and occupational status (Unemployed, homemaker, student, retired, disabled, others/employed), and household income (<$20,000/$20,000–$49,999/$50,000–$74,999/$75,000–$99,999/≥ $100,000) were classified as categorical variables (Table 1).

**Table 1:** Characteristics of the study population stratified by attempted to quit smoking in the past year (*N* = 486).

Characteristics	Total		Attempted to quit	Not attempted to quit
	*N*	% Weighted (95% CI^^^)	% Weighted (95% CI)	% Weighted (95% CI)
*Attempted to quit smoking in the past year*				
Yes	313	60.7 (55.8–65.7)		
No	173	39.3 (34.3–44.2)		
*Perceived neighborhood air quality problems^#^*				
Yes	141	26.1 (21.8–30.3)	74.6 (66.4–82.8)	25.4 (17.2–33.6)
No	345	73.9 (69.7–78.2)	55.9 (49.9–61.8)	44.1 (38.2–50.1)
*Recent advice to quit smoking by a healthcare professional*				
Yes	212	44.9 (39.9–49.9)	59.9 (52.3–67.5)	40.1 (32.5–47.7)
No	274	55.1 (50.1–60.1)	61.5 (54.9–68.1)	38.5 (31.9–45.1)
*Age* (mean [SD^*^])	486	42.3 [18.3]	39.2 [15.9]	48.0 [18.9]
*Sex*				
Female	292	45.5 (40.6–50.3)	69.0 (63.3–74.7)	31.0 (25.3–36.7)
Male	194	54.5 (49.7–59.4)	53.9 (46.3–61.5)	46.1(38.5–53.7)
*Ethnicity/Race*				
Non-Hispanic White	185	51.1 (46.1–56.0)	55.8 (48.0–63.5)	44.2 (36.5–52.0)
Non-Hispanic Black	108	10.5 (8.3–12.7)	57.6 (47.5–67.7)	42.4 (32.3–52.5)
Hispanic	159	31.4 (27.0–35.9)	69.3 (61.5–77.1)	30.7 (22.9–38.5)
Others	34	7.0 (4.6–9.5)	63.1 (45.5–80.7)	36.9 (19.3–54.5)
*Household income*				
< $20,000	105	20.2 (16.2–24.3)	55.2 (44.1–66.3)	44.8 (33.7–55.9)
$20,000–$49,999	190	42.6 (37.5–47.8)	64.1 (56.2–71.9)	35.9 (28.1–43.8)
$50,000–$74,999	80	20.1 (15.7–24.4)	49.1 (36.9–61.2)	50.9 (38.8–63.1)
$75,000–$99,999	43	10.3 (7.1–13.5)	72.3 (57.5–87.2)	27.7 (12.8–42.5)
≥$100,000	29	6.8 (4.1–9.5)	59.6 (39.2–80.1)	40.4 (19.9–60.8)
Marital status				
Single, widowed, divorced, and separated	251	50.4 (45.4–55.4)	53.7 (46.6–60.8)	46.3 (39.2–53.4)
Living as married, Married	235	49.6 (44.6–54.6)	67.9 (61.1–74.7)	32.1 (25.3–38.9)
*Occupational* status				
Unemployed, homemaker, student, retired, disabled, and others	227	48.4 (43.4–53.4)	56.7 (49.3–64.0)	43.3 (36.0–50.7)
Employed	259	51.6 (46.6–56.6)	64.6 (57.9–71.3)	35.4 (28.7–42.1)

^*^SD, Standard deviation. ^CI, Confidence interval. ^#^Perceived neighborhood air quality problems measured by, “Please tell me whether or not each of the following is a problem in your community or neighborhood: Fumes, smells, and smoke from traffic; Fumes, smells, and smoke from industry.” Response options were “yes” and “no.”

### Statistical analysis

The study sample was first screened for outliers and then calibrated against state demographics by ICF International, Inc. (Fairfax, Virginia), using the American Community Survey, Texas (32). Using a three-dimensional raking approach and iterative post-stratification based on sex, age, and four-category race/ethnicity (NHW, NHB, Hispanic, and other), weights were calculated (33). The weighting adjustments help ensure that the weighted sample distributions are similar to the Texas population distribution along key demographic dimensions (age, sex, and race/ethnicity). Statistical analyses were carried out using the survey procedure package in the SAS software (SAS for Windows, version 9.4). We calculated mean and standard deviation for continuous variables, and weighted percentages, and a 95% confidence interval for categorical variables. Multivariable survey logistic regression with survey weights was performed using PROC SURVEYLOGISTIC (34) to examine the factors associated with an attempt to quit smoking outcomes.

## Results

The study population included 486 adults from Texas who smoked in the past 12 months. Table 1 shows the weighted percentages of sociodemographic characteristics, neighborhood problems, and healthcare professionals’ advice to quit smoking stratified by the attempt to quit smoking status. The mean age of the respondents was 42.3 years (standard deviation: + or − 18.3), 54.5% were males, 51.1% were NHW, 42.6% had a household income between $20 k and $49,999, and 51.6% were employed. Air quality problems in the neighborhood were reported by 26.1% of individuals.

There were 313 individuals (60.7%) who attempted to quit smoking in the past year. This proportion was higher among those who reported problems with neighborhood air quality (74.6%) compared to respondents who reported no neighborhood air quality problems (55.9%) (Table 1). In the descriptive analysis (Table 1), quit attempts were higher among females (69%) and individuals living as married or were married (67.9%).

Table 2 reports the results of the survey-weighted logistic regression analysis of factors associated with attempts to quit smoking. We found that individuals who reported having neighborhood air quality problems had 90.6% higher odds of attempting to quit smoking (AOR: 1.906 [1.104–3.289]) than those with no reported air quality problems in the neighborhood. The likelihood of attempts to quit smoking decreased with age (AOR: 0.968 [0.951–0.984]). Moreover, males had 37.1% lower odds of attempts to quit smoking (AOR: 0.629 [0.397–0.995]), and individuals “living or having lived as married” had 87.6% (AOR: 1.876 [1.161–3.033]) higher odds of attempting to quit smoking than those who were single, widowed, divorced, or separated.

**Table 2:** Multivariable weighted survey logistics regression model identifying the association between neighborhood air quality problems and attempts to quit smoking in the past year (*N* = 486).

Characteristics	Categories	AOR	95% CI^^^ Lower limit	95% CI Upper limit	*p* value
Perceived neighborhood air quality problems	Yes	1.906	1.104	3.289	0.021
	No	Ref			
Healthcare professional’s advice to quit smoking	Yes	1.082	0.680	1.721	0.739
	No	Ref			
Age		0.968	0.951	0.984	<0.001
Gender	Male	0.629	0.397	0.995	0.048
	Female	Ref			
Race	Non-Hispanic Black	1.198	0.668	2.148	0.544
	Hispanics	1.253	0.722	2.173	0.422
	Others	1.117	0.453	2.755	0.810
	Non-Hispanic Whites	Ref			
Income	$20,000–$49,999	1.712	0.908	3.227	0.097
	$50,000–$74,999	0.965	0.453	2.055	0.927
	$75,000–$99,999	1.853	0.695	4.939	0.217
	≥ $100,000	1.142	0.406	3.208	0.801
	<$20,000	Ref			
Marital status	Living as Married/Married	1.876	1.161	3.033	0.010
	Single, widowed, divorced, and separated	Ref			
Occupational status	Unemployed, homemaker, student, retired, disabled, and others	1.000	0.605	1.652	1.000
	Employed	Ref			

^^^CI, Confidence interval. ^#^Perceived neighborhood air quality problems measured by, “Please tell me whether or not each of the following is a problem in your community or neighborhood: Fumes, smells, and smoke from traffic; Fumes, smells, and smoke from industry.” Response options were “yes” and “no.”

Other covariates such as healthcare professionals’ advice to quit smoking, and sociodemographic characteristics such as ethnicity/race, income, and employment were not associated with attempting to quit smoking.

## Discussion

In this study, we examined the association between perceived neighborhood air quality problems and attempts to quit smoking among adults. We found that individuals living in areas with higher perceived air quality problems (i.e., exposure to fumes, smells, and smoke from traffic and or industry) were more likely to attempt to quit smoking after adjusting for known predictors such as healthcare professionals’ advice to quit smoking (10), and sociodemographic factors (income, occupation) (8, 9). This suggests that individuals who smoked cigarettes and resided in neighborhoods with air quality problems might attempt to quit plausibly because they perceived having a higher likelihood of adverse health issues given their exposure to poor air quality. In other words, the impact of poor neighborhood air quality on health was high enough to motivate smokers to attempt to quit.

Attempting to quit smoking depends on many factors, such as access to cognitive and pharmacological interventions (4), social support (11), and psychosocial makeup (14, 15). To enable higher attempts to quit smoking in neighborhoods with poor air quality, interventions should focus on raising community awareness of the benefits of quitting smoking, providing training and resources to the community clinic healthcare professionals, and increasing residents’ access to pharmacological and behavioral interventions to help quit smoking. Moreover, policies such as banning smoking in public places, and reducing tobacco retail density may further reduce smoking incentives, triggers, and access (2, 35). Interpreting our study findings in the framework of the transtheoretical model, we can conclude that people living in neighborhoods with poor air quality have a higher likelihood of taking *action* to attempt to quit smoking, which could be enabled by providing adequate resources and implementing policy changes in the region.

The current study also found a significant association between marital status and an attempt to quit, corroborating the existing literature (36, 37). Although our study did not assess the partner’s support in quit attempts, past reports found an increase in quit attempts when there was partner support (11, 38). Since married individuals are more likely to quit smoking, they would benefit if partner education modules were included in quit-smoking interventions. A significant association was also found between age and the attempt to quit, which supports the existing studies (7, 39), suggesting that quit attempts are difficult in older age due to prolonged exposure to psychosocial factors such as nicotine dependence and depression (40).

Furthermore, we found no significant association between healthcare professional advice and an attempt to quit smoking, unlike the existing literature (10, 41). The absence of an association could be because of the suboptimal quality/strength of the advice provided by healthcare professionals to smokers in Texas. A systematic approach to quitting smoking advice by a healthcare professional is essential to motivate patients to attempt to quit (4). Based on the findings of PROJECT TEACH (an initiative in Texas to educate healthcare professionals in community mental health clinics), a lack of training, access to resources, and lack of adequate time were the barriers for healthcare professionals in counseling patients to quit smoking (42).

To our knowledge, this is the first study to assess quit attempts among individuals who smoke and reside in neighborhoods with air quality problems. The current study findings indicate an opportunity for multifold intervention, such as increasing access to evidence-based pharmacological and behavioral interventions for smoking cessation in such neighborhoods. This could be achieved by including spouse or partner education on the quit smoking curriculum, reducing healthcare professionals’ barriers to quitting smoking advice by increasing training and resources, and raising community consciousness on the benefits of quitting smoking through education in these neighborhoods. Medical-academic-community initiatives such as the Be Well™ Communities model were found effective in improving community-level health behaviors by developing community capacity and community-driven coalition. Such a place-based approach could also be used to help increase smoking cessation in neighborhoods with air quality problems (43).

Our study had some limitations. Although perceived neighborhood air quality problems are a common measure used in neighborhood studies (18–20, 44), the self-reported nature of the measure could result in recall and desirability bias. Therefore, future studies should incorporate more objective measures, such as neighborhood-level data on air quality. The survey was conducted online, so the findings can be generalized to a Texas population that has access to the internet and those who have the skills to take online surveys. However, the analysis was conducted using weights to ensure that the weighted sample distributions are similar to the Texas population distribution along age, sex, and race/ethnicity. Moreover, the study did not assess perceptions of smoke generated from sources other than industry and traffic, such as environmental tobacco smoke (ETS) and smoke from natural sources such as wildfire. ETS can lead to second-hand smoking, which can trigger relapse, leading to unsuccessful quit attempts (45). Due to the changing climate, wildfire is an emerging problem in the West Texas area, which will affect the air quality of the region (46), leading to adverse respiratory health (47) and might motivate more people to quit smoking. Therefore, perceptions and roles of ETS and wildfire smoke in quit attempts should be investigated in future studies. Lastly, the data were collected through a cross-sectional survey, so causality could not be established from this study. Nonetheless, a key strength of this study was that the data were collected using a probability sample, and weights were used to ensure the study sample was representative of the Texas adult population.

Perceived neighborhood air quality problems were found to independently predict attempts to quit cigarette smoking in Texas. To enable increased attempts to quit cigarette smoking among individuals living in neighborhoods with poor air quality, such neighborhoods should be supported with tailored and evidence-based interventions to improve community education, social support, and healthcare professionals’ assistance to quit smoking.

## Data availability statement

The raw data supporting the conclusions of this article will be made available by the authors, without undue reservation.

## Ethics statement

The studies involving humans were approved by informed consent was obtained, and the study was approved by the University of Texas MD Anderson Cancer Center’s Institutional Review Board (PA16–0724). The studies were conducted in accordance with the local legislation and institutional requirements. The participants provided their written informed consent to participate in this study.

## Author contributions

MC: Conceptualization, Methodology, Writing – original draft, Writing – review & editing. JF: Conceptualization, Methodology, Writing – review & editing. RY: Data curation, Formal analysis, Writing – review & editing. SS: Conceptualization, Data curation, Methodology, Project administration, Supervision, Writing – original draft, Writing – review & editing.
